# Preoperative AFU Is a Useful Serological Prognostic Predictor for Colorectal Liver Oligometastasis Patients Undergoing Hepatic Resection

**DOI:** 10.7150/jca.31539

**Published:** 2019-08-28

**Authors:** Yuxiang Deng, Yujie Zhao, Wenhua Fan, Jianhong Peng, Xiao Luo, Yiwen Mo, Binyi Xiao, Lin Zhang, Zhizhong Pan

**Affiliations:** 1 Department of Colorectal Surgery, State Key Laboratory of Oncology in South China, Collaborative Innovation Center for Cancer Medicine, Sun Yat-sen University Cancer Center, Guangzhou, China.; 2 Department of Ultrasound, State Key Laboratory of Oncology in South China, Collaborative Innovation Center for Cancer Medicine, Sun Yat-sen University Cancer Center, Guangzhou, China.; 3 Department of Nuclear Medicine, State Key Laboratory of Oncology in South China, Collaborative Innovation Center for Cancer Medicine, Sun Yat-sen University Cancer Center, Guangzhou, China.; 4 Department of Clinical Laboratory, State Key Laboratory of Oncology in South China, Collaborative Innovation Center for Cancer Medicine, Sun Yat-sen University Cancer Center, Guangzhou, China.

**Keywords:** preoperative AFU, CLOM, prognosis, OS

## Abstract

**Background**: Preoperative alpha-l-fucosidase (AFU) has been used as a diagnostic biomarker for several cancers, but its role as a prognostic predictor in colorectal cancer liver oligometastasis (CLOM) patients after radical surgery has not been well defined. This study aimed to investigate the prognostic significance of preoperative serum AFU for CLOM patients after hepatic resection.

**Methods**: A retrospective data set was collected to evaluate the prognostic value of preoperative AFU in CLOM patients after radical hepatic resection. A total of 269 patients with histopathologically confirmed CLOM were enrolled. The optimal cut-off value of preoperative AFU was determined using X-tile software. Univariate and multivariate analyses were used to identify the prognostic significance of preoperative serum AFU.

**Results**: The X-tile software showed that the optimal cut-off value of preoperative AFU was set at 30.8 U/L. Patients with preoperative AFU≤30.8 and >30.8 were classified into high and low AFU groups, respectively. Female patients and those with a single liver metastasis had a higher tendency to have a preoperative AFU≤30.8 U/L; patients with lower clinical risk score (CRS) were more likely to have AFU >30.8 U/L than patients with higher CRS. The results showed that preoperative AFU was an independent prognostic factor for overall survival (OS) (P=0.041). Patients with a preoperative AFU≤30.8 U/L had a lower OS rate than those with AFU>30.8 U/L. Furthermore, for patients with lower CRS scores (0-2), the tendency clearly showed that patients with higher preoperative AFU had a better prognosis (P=0.029).

**Conclusions**: Higher preoperative serum AFU can predict better survival in CLOM patients after hepatic resection, especially for CLOM patients with lower CRS scores.

## Introduction

The global burden of cancer worldwide using the GLOBOCAN 2018 estimated that colorectal cancer (CRC) was the fourth most common cancer in incidence and the second leading cause of cancer death 1. The liver is the most frequent site of metastatic disease. For patients with colorectal cancer liver metastasis (CRLM), hepatic resection is the main curative choice 2. In the latest European Society for Medical Oncology (ESMO) guidelines, there are two categories for metastatic CRC: oligometastatic disease (OMD) and widespread systemic disease 3. OMD is a state of metastatic disease that is limited in total disease burden, according to the limited amount of clinical or radiographic evidence 4, 5. OMD represents a disease state that exists in a transitional zone between localized and widespread systemic diseases. It has a genuine potential for cure when patients receive complete R0 resection of their metastases 6, 7. Because the prognoses of these two types of patients are quite different, the treatment strategies are also different. To date, few studies have highlighted the clinical survival of colorectal liver oligometastases (CLOM) patients who undergo curative resection. Therefore, identifying efficient prognostic factors for CLOM patients is urgently needed to screen for high-risk subgroups and to develop adequate therapeutic interventions for maximum therapeutic effectiveness.

α-L-fucosidases (AFU), a lysosomal enzyme in nature^8^, is widely expressed in many types of cells. AFU catalyses the hydrolytic cleavage of terminal fucose residues, but its physiological functions are not completely understood. Previous observations indicated that human AFU is downregulated in highly aggressive human tumours, such as neuroblastomas 9, breast cancer 10, and colorectal cancers 11. Serum and salivary AFU activity were significantly higher in OPC and oral cancer patients compared to controls 12. Since elevated fucose levels are preferentially expressed in metastatic foci versus primary tumours 13-15, it has been suggested that the study of altered fucose in tumour cells could be useful in searching new treatment targets 16. AFU has also been used as a biomarker for the early diagnosis of hepatocellular carcinoma (HCC) 17, as well as for its potential usefulness in clinical diagnosis 18-21. As for its prognostic value, preoperative AFU is also a powerful prognostic indicator for HCC 22.

For CRC, previous study had revealed the prognostic predicting role of serum AFU activity in non-metastatic CRC patients 23. However, to our knowledge, no studies have focused on the predictive role of preoperative AFU as an effective indicator of the prognosis of CLOM patients undergoing curative hepatic resection. In addition, the optimal cut-off value of AFU for the prediction of oncologic outcomes remains controversial. In this study, we aimed to identify the optimal AFU cut-off value and to investigate the prognostic impact of preoperative AFU in patients with CLOM who underwent curative hepatic resection.

## Materials and Methods

### Patient population

**Figure 1:**
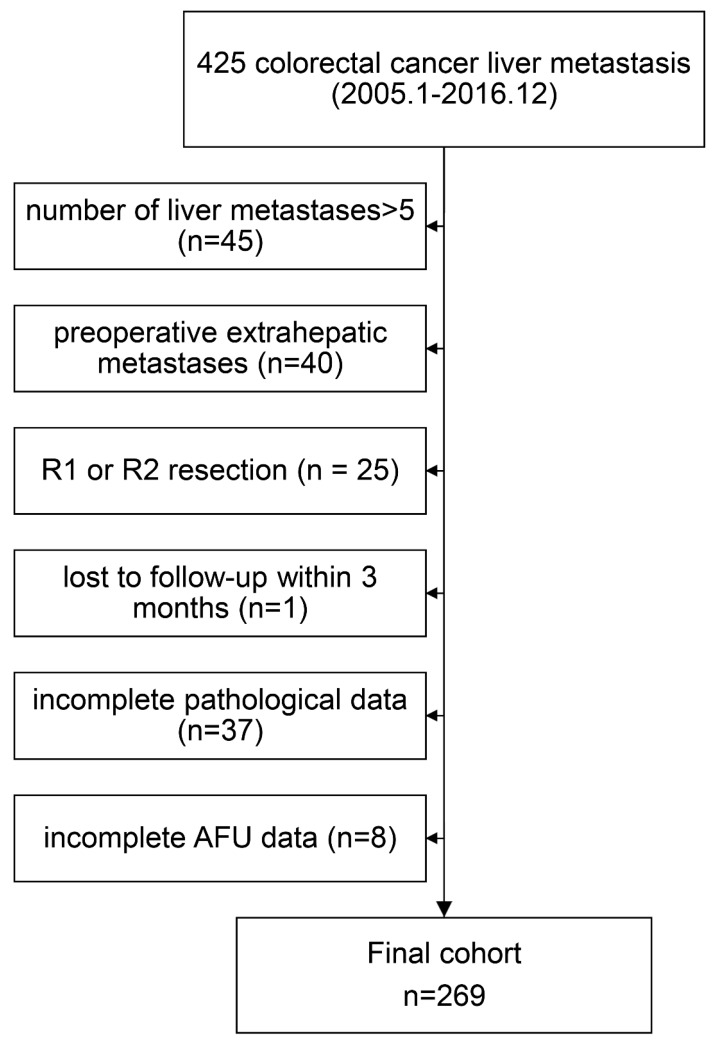
Flow chart of the analysed patients.

We retrospectively examined data from 425 colorectal cancer liver metastasis patients who underwent curative resection at Sun Yat-sen University Cancer Center from January 2005 to December 2016. The enrolled patients met the following inclusion criteria: (1) histologically confirmed adenocarcinoma; (2) colorectal liver oligometastases (≤5 metastases); (3) no preoperative extrahepatic metastases; and (4) R0 resection both for primary lesions and metastases. We excluded 156 patients based on the following exclusion criteria: number of liver metastases>5 (n = 45); preoperative extrahepatic metastases (n=40); R1 or R2 resection (n = 25); loss to follow-up within 3 months (n=1); incomplete pathological data (n=37); and incomplete AFU data (n=8). The final cohort thus consisted of 269 patients (Figure 1). This study was conducted with the approval of the Institute Research Ethics Committee of the Sun Yat-Sen University Cancer Center.

### Clinical risk score (CRS)

The clinical risk score (CRS) was used to predict recurrence after hepatic resection for metastatic CRC patients, which was also used as a stratifying factor in our study. The following five clinical criteria were chosen as the criteria for CRS: nodal status of primary, disease-free interval from the primary to discovery of liver metastases of <12 months, number of tumours >1, preoperative carcinoembryonic antigen (CEA) level >200 ng/ml, and size of the largest tumour >5 cm. Each criterion was assigned one point according to the total score of CRS. Patients were divided into two groups: a low-risk group (score: 0-2) and a high-risk group (score: 3-5) 24.

### Patient treatments

Primary tumours were staged according to the seventh edition of the UICC-TNM staging system for colorectal cancer. Preoperative tumour status was evaluated using a combination of colonoscopy, computed tomography (CT), ultrasonography, magnetic resonance imaging (MRI), and positron emission tomography. The decision and timing of surgical resection of the primary tumour was based on clinical assessment by a surgical oncologist in conjunction with a multidisciplinary team. The extent and nature of hepatic surgery were similarly individualized for each patient based on rigorous clinicopathological evaluation.

### Serum biological factors

Patients received routine serum tests for biochemical detection between 7 and 10 a.m. within 7 days before the hepatic operation, and samples were centrifuged at 3500 g/min for 8 min to allow serum separation. The levels of AFU were measured in serum using a Hitachi 7600 automatic biochemical analyser (Hitachi High-Technologies, Tokyo, Japan). CEA and cancer antigen 19-9 (CA19-9) were detected using an electrochemiluminescence immunoassay system (Elecsys 1601; Roche, Basel, Switzerland) according to the manufacturer's instructions, and the cut-off values for CEA and CA19-9 were 5 ng/mL and 37 U/mL, respectively.

### Follow-up

The follow-up protocol included evaluations every 3 months for the first 2 years after surgery, every 6 months for the third to fifth years and once every year thereafter. Evaluations at each visit included obtaining a complete blood count, evaluations of CEA and CA199 levels, and a physical examination. Chest radiography, abdominal and pelvic CT, colonoscopy, and pelvic MRI were conducted every year. Overall survival was defined as time from liver resection to death or last follow-up. Patients alive at the last follow-up date were regarded as randomly censored. Follow-up was terminated in September 2018.

### Statistical analysis

The cut-off value for AFU was assessed using X-tile 3.6.1 software (Yale University, New Haven, CT, USA), identified from the minimum P value according to OS. Statistical analyses were performed using SPSS 20.0 software (IBM, Chicago, IL, USA) and GraphPad Prism 7 software (GraphPad Software, Inc., San Diego, CA, USA). We compared categorical variables using the chi-square (χ2) test. The Kaplan-Meier method was used to estimate the survival rates for the different groups, and differences in survival were compared with the log-rank test. A multivariate Cox proportional hazards analysis was performed using variables with P values less than 0.05 in univariate analysis. P<0.05 was considered statistically significant.

## Results

### Patient characteristics and optimal cut-off value of AFU

**Figure 2:**
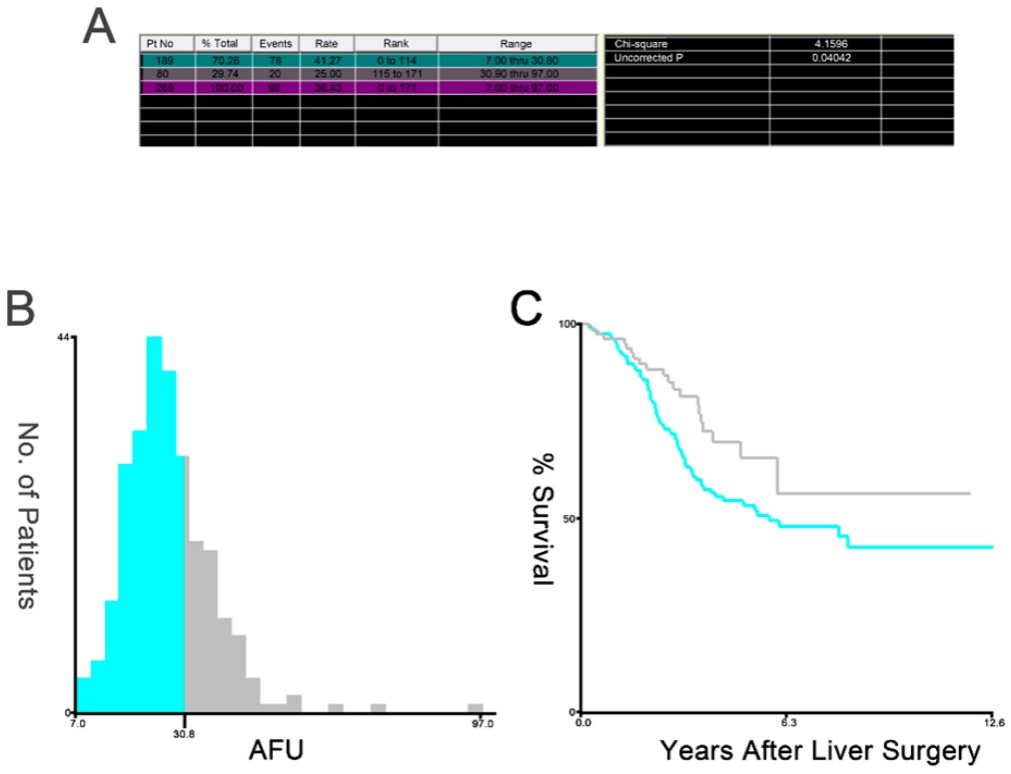
X-tile plots of the AFU and the OS of patients with CLOM who underwent curative resection. X-tile plots showing χ2 values with cut-off points to generate the low- and high-AFU subgroups. (A) The optimal cut-off value of the AFU was 30.8 at the maximum χ2 value of 4.1596. (B) Histogram of the entire cohort divided into low-AFU and high-AFU subgroups according to the optimal cut-off value of 30.8. Blue bars represent the low-AFU group, and grey bars represent the high-AFU group. (C) Kaplan-Meier plot of OS in groups stratified using the optimal cut-off value of AFU. Blue curves represent the low-AFU group, and grey curves represent the high-AFU group.

Table 1 shows the patients' clinical and pathological characteristics. The mean age of all patients was 56±12 years, and 66.2% of the patients were male. X-tile software was used to determine 30.8 as the optimal cut-off value for AFU (Figure 2). Based on the serum AFU level, 189 patients were classified into the low-AFU (AFU≤30.8) group, and 80 patients were classified into the high-AFU (AFU>30.8) group. The associations between AFU level and various clinicopathological features are shown in Table 2. Among all 269 patients, there were no significant differences in the distribution of age, tumour location or TN stage between the two groups. Moreover, there were no significant differences in the histological grade, liver metastases tumour size, hepatic resection timing, or CEA level and CA199 level of the patients in the low- and high-AFU groups. Females, patients who had single liver metastasis and those with higher CRS were more frequently observed in the low-AFU group (P=0.046; P=0.041; and P=0.007, respectively).

### Association between AFU and survival

**Figure 3:**
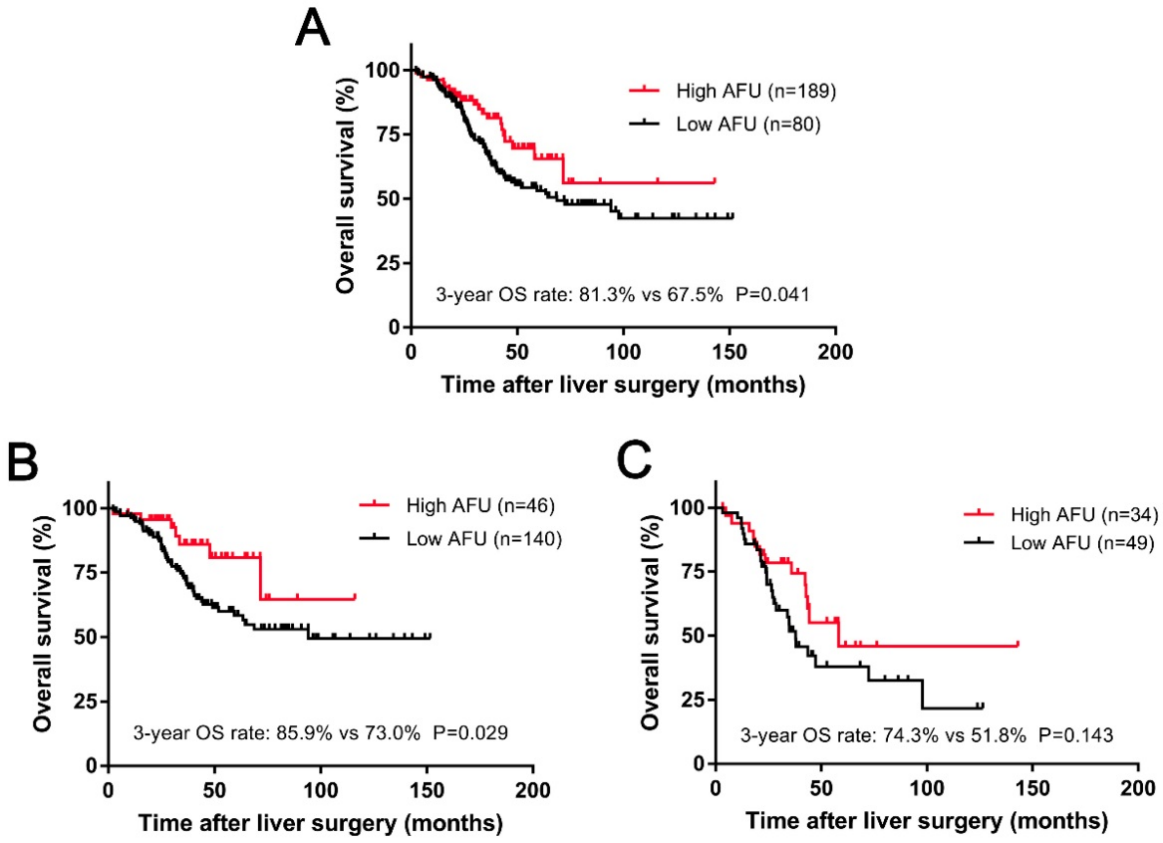
Kaplan-Meier curves for 3-year OS (A) based on the AFU. Kaplan-Meier curves for 3-year OS (B) based on the AFU in subgroups with low CRS (score: 0-2) and for 3-year OS (C) based on the AFU in subgroups with high CRS (score: 3-5).

The median follow-up time for all patients was 36.7 months (range 2.3-151.7 months) after liver resection. During that period of time, 98 (36.4%) patients were ultimately dead because of tumour progression. An obvious difference was observed in patients with different levels of preoperative AFU. Kaplan-Meier analysis indicated that the 3-year OS rate in the low-AFU group was significantly lower than that in the high-AFU group (67.5% vs 81.3%, P=0.041; Figure 3A).

### Univariate and multivariate analyses of prognostic factors

The univariate and multivariate analyses are summarized in Table 3. The univariate analyses showed that OS was significantly associated with primary tumour site (P=0.003), N stage (P=0.002), number of liver metastases (P=0.011) and AFU (P=0.043). In addition, Cox multivariate analysis showed that the following were independent poor prognostic factors for OS: primary tumour in rectum (P<0.001), advanced N stage (P<0.001), multiple liver metastases (P=0.001), and operative AFU level≤30.8 (P=0.001).

### Prognostic analysis in patients with different AFU levels in accordance with CRS

In our study, CLOM patients were divided into two subgroups according to CRS. One hundred and eighty-six (69.1%) patients belonged to the CRS-low group (score: 0-2), and 83 (30.9%) patients were in the CRS-high group (score: 3-5). We conducted a survival analysis based on the CRS group, and an interesting result was found. For patients with low CRS, a significant deterioration in survival was observed for patients with low AFU compared with patients with high AFU (3-year OS: 85.9% vs 73.0%, P=0.029; Figure 3B). However, for patients with a higher CRS (score: 3-5), despite the tendency towards survival diversity, there was no significant difference in the 3-year OS rate between high AFU and low AFU (P=0.143) (Figure 3C).

## Discussion

AFU was reported to be a lysosomal protein 25, which catalyses the hydrolytic cleavage of terminal alpha-L-fucose residues in glycoproteins and glycolipids 26. Alpha-L-fucose-containing molecules are key aspects for the progression of tumours, including haematogenous metastasis 16, tumour invasion through extracellular matrices 27, and upregulation of Notch signalling^28^, ^29^, with implications for the epithelial-to-mesenchymal transition (EMT) and activation of cancer stem cells. AFU has been previously studied for its diagnostic value in several cancers. For example, AFU has been proven to be a potential diagnostic marker for HCC 19, 21 and CRC 30. Another study showed that AFU in combination with CD26 can form a molecular model for diagnosis, especially for non-disseminated CRC 31. The combination of AFU and TCH can have better diagnostic accuracy in comparison with their individual detection ability, which is helpful for differential diagnosis between malignant and non-tuberculous benign ascites 32. In our study, we found that increased preoperative AFU in peripheral blood can predict better survival in CRLM patients undergoing hepatic resection. In a previous study, it was demonstrated that AFU can remove α-L-fucose from oligosaccharide sites on metastatic cancer cells 33. Therefore, based on the results of our study, we hypothesized that high AFU expression could decrease the expression of fucose-containing molecules on the surface of colorectal cancer cells, thereby significantly inhibiting tumour cell invasion. That could explain the results of our study regarding why higher AFU can predict a better outcome. It might be expected that the enzyme AFU, which is involved in the removal of fucose-containing glycoproteins and glycolipids, could play an important role in the maintenance of the fucose content of aberrantly fucosylated glycoconjugates.

In a number of studies related to AFU, researchers used different methods to determine its cut-off value 34, 35. In our study, the cut-off value for AFU was identified using X-tile software. This tool is used to assess the biological relationships between a biomarker and clinical outcome and to determine the cut-off points based on marker expression level 36. This statistical method has been shown to be effective in a number of studies 35, 37.

In particular, we found that AFU can be an efficient prognostic predictor for CRS low (score: 0-2) patients, not CRS high (score: 3-5) patients. The CRS introduced by Fong et al has been used in predicting recurrence after hepatic resection for metastatic colorectal cancer patients undergoing resection of liver metastases 24. In our study, we performed Kaplan-Meier analysis on the survival of patients stratified by groups with low CRS risk and high CRS risk. In the subgroup with low CRS risk, higher AFU was associated with favourable OS. However, in subgroups with high CRS risk, the result was not significant. This may be because patients with low-risk CRS have longer survival, so the relationship between AFU and prognosis can be observed. Our results suggest that for patients with low recurrence risk, there are still factors to which we should pay attention. In clinical applications for patients with low CRS, treatment should depend on the level of AFU. If patients belong to the high-AFU group (>30.8), they can consider undergoing less strict follow-up. However, larger and more objective prospective studies are needed to guide clinical decision making.

The lysosomal enzyme AFU coding gene FUCA-1 has been widely studied 38. In breast cancer, it has been proven that FUCA-1 has independent prognostic value. Low FUCA-1 expression correlated with a significantly shorter relapse-free survival (RFS) as well as OS, while FUCA-1 overexpression was associated with a relatively good outcome 39. Furthermore, decreased expression of the FUCA-1 gene was also found in human colorectal carcinomas compared with normal counterparts. A gradual decrease in FUCA-1 expression was observed along with the progression of CRC from earlier to advanced stages 11. In highly aggressive and metastatic human tumours, the FUCA-1 gene is downregulated, possibly because its inactivation disturbs the fucosylation of proteins involved in cell adhesion, migration and metastases. Yuan et al hypothesized that a decrease in fucose content might alter the biological behaviour of breast cancer cells and, in particular the interaction among tumour cells, the ECM and endothelial cells, yielding new information for the diagnosis and treatment of metastases 16. Furthermore, the expression of the FUCA-1 gene has been reported to be directly controlled by p53 40, 41. Mutated p53 has been associated with lower expression of FUCA-1 in human thyroid cancer cell lines 41.

However, this study has some limitations. First, it was a retrospective single-institution study. Several diseases, such as hepatitis and cirrhosis, which can affect the value of AFU, were not screened. This may lead to a selection bias. Second, numerous studies have shown different cut-off values for AFU level, so this requires further validation. Finally, the short duration of follow-up time was insufficient to evaluate longer survival outcomes.

In conclusion, patients with low AFU have various disadvantages in clinical background that can adversely affect long-term outcomes after hepatic resection. Particularly, for patients with lower CRS, AFU can predict postoperative survival more accurately. Higher preoperative AFU may become a convincing marker to estimate better survival outcomes for CLOM patients undergoing hepatic resection.

## Figures and Tables

**Table 1:** Clinicopathologic features of patients involved in this study.

Characteristics		No. (%)
**Age (years)**	≤60	159 (59.1)
	>60	110 (39.1)
**Gender**	Female	91 (33.8)
	Male	178 (66.2)
**Primary tumor site**	Colon	172 (63.9)
	Rectum	97 (36.1)
**T stage**	1	1 (0.4)
	2	25 (9.3)
	3	148 (55.0)
	4	95 (35.3)
**N stage**	0	115 (42.8)
	1	97 (36.1)
	2	57 (21.2)
**Histological grade**	Well/moderate	237 (88.1)
	Poor	32 (11.9)
**Liver metastases tumor size (cm)**	≤2.3	136 (50.6)
	>2.3	133 (49.4)
**Liver metastases number**	Single	140 (52.0)
	Multiple	129 (48.0)
**Hepatic resection timing**	Synchronous	161 (59.9)
	Metachronous	108 (40.1)
**CEA (ng/ml)**	≤5	117 (43.5)
	>5	152 (56.5)
**CA199 (U/ml)**	≤37	205 (76.2)
	>37	64 (23.8)
**CRS**	0-2	186 (69.1)
	3-5	83 (30.9)
**AFU (U/L)**	≤30.8	189 (70.3)
	>30.8	80 (29.7)

Abbreviations: CEA, carcinoembryonic antigen; CA19-9, cancer antigen 19-9; CRS, clinical risk score; AFU, alpha-l-fucosidase.

**Table 2:** Relationships between AFU and patient characteristics

Characteristics	AFU (U/L) (n=269)		
	≤30.8 (n=189), n (%)	>30.8 (n=80), n (%)	P value
**Age (years)**			
≤60	105 (55.6)	54 (67.5)	0.069
>60	84 (44.4)	26 (32.5)	
**Sex**			
Female	71 (37.6)	20 (25.0)	0.046
Male	118 (62.4)	60 (75.0)	
**Primary tumor site**			
Colon	123 (65.1)	49 (61.2)	0.550
Rectum	66 (34.9)	31 (38.8)	
**T stage**			
1-3	125 (66.1)	49 (61.2)	0.443
4	64 (33.9)	31 (38.8)	
**N stage**			
0	85 (45.0)	30 (37.5)	0.257
1-2	104 (55.0)	50 (62.5)	
**Histological grade**			
Well/moderate	165 (87.3)	72 (90.0)	0.532
Poor	24 (12.7)	8 (10.0)	
**Liver metastases tumor size (cm)**			
≤2.3	98 (51.9)	38 (47.5)	0.514
>2.3	91 (48.1)	42 (52.5)	
**Liver metastases number**			
Single	106 (56.1)	34 (42.5)	0.041
Multiple	83 (43.9)	46 (57.5)	
**Hepatic resection timing**			
Synchronous	118 (62.4)	43 (53.8)	0.184
Metachronous	71 (37.6)	37 (46.2)	
**CEA (ng/ml)**			
≤5	84 (44.4)	33 (41.2)	0.629
>5	105 (55.6)	47 (58.8)	
**CA199 (U/ml)**			
≤37	149 (78.8)	56 (70.0)	0.120
>37	40 (21.2)	24 (30.0)	
**CRS**			
0-2	140 (74.1)	46 (57.5)	0.007
3-5	49 (25.9)	34 (42.5)	

Abbreviations: AFU, alpha-l-fucosidase; CEA, carcinoembryonic antigen; CA19-9, cancer antigen 19-9; CRS, clinical risk score.

**Table 3:** Univariate and multivariate analyses of the factors influencing OS by Cox proportional hazard model

	Univariate analysis		Multivariate analysis	
	HR (95% CI)	P value	HR (95% CI)	P value
**Age (year)**				
≤60 vs. >60	1.374 (0.923-2.048)	0.118		
**Gender**				
Female vs. Male	1.224 (0.799-1.876)	0.353		
**Primary tumor site**				
Colon vs. Rectum	1.837 (1.235-2.732)	0.003	2.174 (1.448-3.364)	<0.001
**T stage**				
1-3 vs. 4	1.096 (0.746-1.611)	0.639		
**N stage**				
0 vs. 1-2	1.955 (1.271-3.005)	0.002	2.414 (1.552-3.754)	<0.001
**Histological**				
Well/moderate vs. Poor	1.472 (0.861-2.518)	0.157		
**Liver metastases tumor size (cm)**				
≤2.3 vs. >2.3	1.476 (0.987-2.207)	0.058		
**Liver metastases number**				
Single vs. Multiple	1.685 (1.128-2.518)	0.011	2.027 (1.343-3.058)	0.001
**Hepatic resection timing**				
Synchronous vs. Metachronous	1.297 (0.871-1.930)	0.200		
**CEA (ng/ml)**				
≤5 vs. >5	1.245 (0.829-1.870)	0.290		
**CA199 (U/ml)**				
≤37 vs. >37	1.051 (0.659-1.678)	0.834		
**AFU (U/L)**				
≤30.8 vs. >30.8	0.601 (0.367-0.984)	0.043	0.438 (0.264-0.728)	0.001

Abbreviations: OS, overall survival; HR, hazard ratio; CI, confidence interval; CEA, carcinoembryonic antigen; CA19-9, cancer antigen 19-9; AFU, alpha-l-fucosidase.

## Abbreviations

AFU: alpha-l-fucosidase
CLOM: colorectal cancer liver oligometastasis
CRS: clinical risk score
OS: overall survival
CRC: colorectal cancer
CRLM: colorectal cancer liver metastasis
ESMO: European Society for Medical Oncology
OMD: oligometastatic disease
HCC: hepatocellular carcinoma
CEA: carcinoembryonic antigen
CT: computed tomography
MRI: magnetic resonance imaging
CA199: carbohydrate antigen 19-9
EMT: epithelial-to-mesenchymal transition
HR: hazard ratio
CI: confidence interval
